# Detection of pit and fissure sealant microleakage using quantitative light-induced fluorescence technology: an in vitro study

**DOI:** 10.1038/s41598-024-59651-x

**Published:** 2024-04-20

**Authors:** Sang-Mi Nam, Hye-Min Ku, Eun-Song Lee, Baek-Il Kim

**Affiliations:** 1 Department of Dental Hygiene, SahmYook Health University, Seoul, Republic of Korea; 2 Department of Preventive Dentistry & Public Oral Health, BK21 FOUR Project, Yonsei University College of Dentistry, 50-1 Yonsei-ro, Seodaemun-gu, Seoul, 120-749 Republic of Korea

**Keywords:** Dental microleakage, Optical imaging, Optical devices, Pit and fissure sealants, Quantitative light-induced fluorescence, Risk factors, Signs and symptoms

## Abstract

This in vitro study aimed to evaluate the feasibility of quantitative light-induced fluorescence (QLF) technology for detecting the presence and severity of microleakage of pit and fissure sealants. The areas of interest (AOIs) were 160 pits and fissures of 40 extracted permanent teeth. Fluorescent images were acquired using a QLF device, and the maximum fluorescence loss ΔF_max_ of each AOI was analyzed. After staining and cross-sectioning of the teeth, histological dye penetration was scored on a scale of 0 to 3. The relationship between ΔF_max_ and microleakage depth was analyzed, and the areas under the curve (AUCs) were calculated. The │ΔF_max_│ increased as microleakage depth increased. The ΔF_max_ values of microleakage areas showed a strong significant correlation with the histological scores of dye penetration (r = − 0.72, P = 0.001). AUC analysis showed a high diagnostic accuracy for microleakage depth (AUC = 0.83–0.91). The highest AUC of 0.91 was found when differentiating the outer half microleakage of the sealant (histological score 0 vs. 1–3). QLF technology is effective in assessing the presence and severity of microleakage, suggesting its potential for noninvasive detection and monitoring of sealant microleakage in clinical settings.

## Introduction

Microleakage is defined as the passage of bacteria, oral fluids, molecules, or ions through minute gaps that are difficult to observe with the naked eye, occurring between the tooth and dental restoration^1^. According to a previous study, microleakage around pit and fissure sealants can cause fatigue and aging due to temperature changes in the oral environment ranging from 5 to 55 °C^2^. Inexperienced professionals may inadequately control tooth surface moisture causing the surface not to be completely dry. This mistake may lead to inadequate penetration, potentially compromising marginal sealing^3,4^. Microleakage may occur owing to the insufficient ability of conventional moisture control methods (e.g., rubber dam isolation) alone to achieve optimal sealant retention^5,6^.

Consequently, recent studies have explored various methods to enhance the retention of sealants^7–9^. To prevent microleakage during sealant application, meticulous cleaning and initial sealing are crucial. The conventional pretreatment method, involving etching using 35% phosphoric acid and cleaning processes prior to sealant application, reportedly causes difficulties in completely removing organic substances from the tooth surface^7,8^. Several studies have explored pretreatment with sodium hypochlorite (NaOCl) before phosphoric acid etching to overcome these limitations and improve material retention^7,8^. In a randomized clinical trial, pretreatment with NaOCl before etching reportedly can facilitate enamel deproteinization, leading to improved retention, and after 1 year of follow-up, the marginal integrity was improved compared with that in the group using phosphoric acid alone^9^. Various pretreatment methods have been reported to enhance the retention of materials and reduce the likelihood of microleakage around sealants.

Another crucial aspect for improving retention is the inherent initial sealing ability of the material, particularly when applying sealants to teeth with narrow and deep fissures. The initial sealing quality may differ depending on the flow capacity of the sealing material^10^. Previous studies have reported that the initial sealing ability was enhanced when composite resin was mixed with resin infiltrant. This was attributed to the effectiveness of low-viscosity resin in penetrating through capillary forces^11,12^. Numerous studies have reported that materials with lower viscosity exhibit superior marginal adaptation^13,14^. Thus, it may be possible to prevent microleakage and ultimately contribute to improving sealant retention by utilizing low-viscosity sealing materials to enhance initial sealing capability.

While various factors contribute to restoration-material failure, microleakage has been highlighted as a significant factor^15^. Microleakage can lead to several problems, such as marginal discoloration of the restoration, marginal fractures, recurrent decay, tooth sensitivity, and dimensional changes, ultimately leading to the need for retreatment^16^. Pit and fissure sealants have been reported to be effective in preventing dental caries and inhibiting the progression of non-cavitated carious lesions by sealing the occlusal surfaces of teeth using resin or glass ionomer cement^17–19^. To prevent dental caries, the pit and fissure sealants should remain well bonded to the enamel surface and effectively prevent microleakage in the early stages^20,21^. Therefore, it is important to objectively and accurately diagnose the subtle changes that occur after the use of pit and fissure sealants in clinical practice.

Partial microleakage can affect sealant retention. Microleakage can occur because of the inaccurate application of the material during the sealant treatment process, and the physical characteristics of the sealant may also lead to microleakage^22^. If the sealant is applied improperly, microleakage may occur because of daily oral function, potentially leading to secondary caries below the microleakage^23^. In addition, the ability of a sealant to flow microscopically into narrow pits and fissures of teeth is important, which is why some sealant products do not contain fillers^14^. This causes the sealant to have relatively weak physical properties, which promote partial microleakage. Over time, this can lead to the development of dental caries in the subsurface^24^. Therefore, the detection of partial microleakage caused by various factors is essential for maintaining the sealant can prevent the development and rapid progression of secondary caries.

Conventional methods for detecting sealant microleakage involve assessing the boundary surface of the sealant using dye penetration or identifying secondary carious lesions caused by microleakage via radiographs. Additionally, traditional techniques such as tactile inspection of the sealant-to-tooth interface and visual detection of color changes in secondary carious lesions are commonly employed in clinical settings. However, these methods have low validity and reliability, largely because precise measurements are difficult to obtain due to the narrow gap between the sealant and enamel^25,26^. Various studies have evaluated microleakage using different optical devices; representative devices for optical assessment include swept-source optical coherence tomography (SS-OCT) and DIAGNOdent^27,28^. A previous study has explored the use of DIAGNOdent to objectively identify microleakage in sealants, thereby overcoming the limitations of traditional inspection techniques^29^. Several studies have reported the use of SS-OCT for detecting defects at the tooth-restoration interface^28,30,31^. SS-OCT, which utilizes light in the near-infrared range (1260–1360 nm), can provide cross-sectional images of tissues; however, its penetration depth is limited. SS-OCT has limitations in imaging deeper regions of biological tissues because of the limited penetration depth caused by the characteristics of the light source^31^. Another optical device for the detection of sealants is the laser fluorescence device DIAGNOdent. This device utilizes 655 nm laser fluorescence, demonstrating high accuracy in detecting secondary caries around the sealant. However, false-positive reactions have been reported for restorative materials^27^.

Quantitative light-induced fluorescence (QLF) is a representative optical technique that utilizes the autofluorescence of teeth and bacteria^32,33^. This technique involves illuminating the teeth with blue-violet visible light at a wavelength of 405 nm. It quantifies the autofluorescence emitted by sound enamel or the presence of bacterial porphyrin fluorescence, allowing the quantification of dental caries or other lesions^21,34–39^. A study reported that QLF was employed to monitor sealant over a long-term period after sealant application, detect whether dental caries progressed severely, and predict the need for sealant repair^40^. Another study applied fluoride to white spots and monitored the changes over an extended period using QLF^41^. Similar to these previous studies, it is essential to assess the utility of QLF in detecting microleakage around sealants and evaluating changing patterns through the continuous monitoring of microleakage. A few studies have applied QLF to detect and monitor caries around pit and fissure sealants, evaluating the performance of this technique in comparison with that of clinical examination methods^40,42^. Furthermore, QLF can detect metabolic products such as porphyrins produced by oral bacteria through red autofluorescence. This allows the observation of bacterial structures, including plaques and calculus, as well as tissues infiltrated by bacteria, indicating red fluorescence^43,44^. QLF can easily detect the occurrence of secondary caries around restorations^45–47^. In the case of microleakage occurring in the pit and fissure sealant, bacteria infiltrating the leakage can be observed using the red fluorescence of QLF. A study evaluating the detection of secondary caries around permanent tooth sealants using QLF reported that it had higher validity in assessing secondary caries in enamel and dentin than visual inspection and radiographic examination^48^. Furthermore, artificial carious lesions formed around resin restorations were evaluated using QLF. QLF can detect incipient secondary caries, but distinguishing it from staining is challenging^49^. However, research assessing the validity of QLF measurements in relation to the depth of microleakage in pits and fissure sealants of permanent teeth is limited.

The present in vitro study was to evaluate the feasibility of detecting the presence and severity of microleakage in pit and fissure sealants using QLF technology. The null hypothesis of this study was that there would be no significant relationship between the fluorescence variable (ΔF_max_) obtained from QLF technology and the actual depth of microleakage (µm) from the pit and fissure sealant. For this purpose, the maximum fluorescence loss parameter (ΔF_max_) and the histological dye penetration score were compared.

## Results

### Sample distribution

This study examined 160 sites, comprising four areas from each of 40 enrolled teeth, with four teeth excluded due to damage incurred during the experiment (Table 1). Following dye penetration, histological evaluations revealed that approximately 22% of the tooth sections exhibited no microleakage (score 0), whereas 28% received a score of 3, indicating complete dye penetration into the base of the pit and fissure.

**Table 1:** Distribution of ΔF_max_ according to the dye penetration histological score after microleakage formation.

Dye penetration histological score	N (distribution%)	Fluorescence variable
		│ΔF_max_│
0	35 (21.9%)	7.3 ± 2.5^a^
1	39 (24.4%)	11.2 ± 4.4^b^
2	41 (25.6%)	15.9 ± 4.6^c^
3	45 (28.1%)	19.4 ± 7.5^d^

All values are expressed as mean ± standard deviations. │ΔF_max_│ means absolute value of maximum fluorescence loss. Different letters within the same column indicate significant differences between the groups using Games–Howell post hoc analysis at a = 0.05.

### Differences in ΔF_max_ according to the depth of sealant microleakage

**Figure 1:**
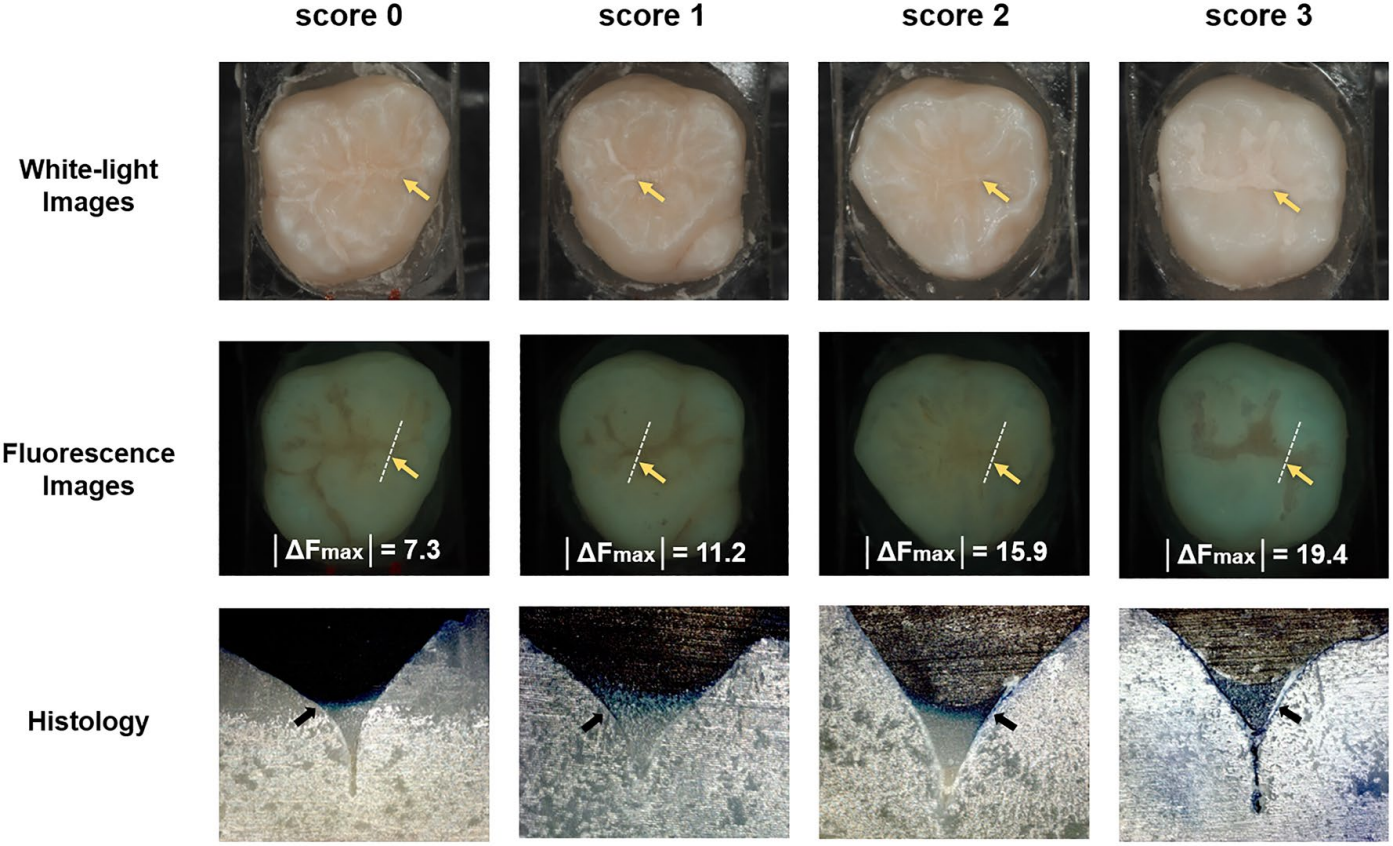
Representative fluorescence images and magnified microscopy images (×50) of the specimens according to the histological scores of dye penetration. The analysis area is indicated by yellow arrows, and the microleakage is indicated by black arrows. The white dashed lines in the fluorescent images represent the cross-section and analyzed regions.

As the depth of microleakage in the pit and fissure sealants increased, the absolute value of ΔF_max_ measured at the occlusal surface for areas with sealant microleakage showed a significant increase (P = 0.004; Table 1, Fig. 1). The ΔF_max_ values of the microleakage areas showed a significantly strong correlation with increasing histological scores of dye penetration (r = − 0.72, P = 0.001). The average ΔF_max_ value for each dye penetration score significantly differed among all scores (P = 0.004). Particularly, the │ΔF_max_│ value for the most severe microleakage (score 3: 19.4) was approximately 2.6 times higher than that for the intact condition with no microleakage (score 0: 7.3).

### Diagnostic capability to distinguish the severity of microleakage using ΔF_max_

To assess the diagnostic accuracy of the fluorescence variable ΔF_max_ in relation to the depth of microleakage in pit and fissure sealants, sensitivity, specificity, and area under the curve (AUC) were calculated at the optimal cutoff point, based on the analysis of three diagnostic thresholds (Table 2). The AUC for ΔF_max_ demonstrated a high level of diagnostic accuracy, ranging from 0.83 to 0.91 across all diagnostic thresholds for detecting microleakage (P = 0.001). Specifically, when distinguishing microleakage beyond the outer half of the pit and fissure sealant (histological scores 1–3) from a lack of microleakage (histological score 0), a ΔF_max_ value of 10.10 yielded a sensitivity of 0.82, a specificity of 0.91, and the highest AUC value of 0.91.

**Table 2:** Area under the ROC curve, optimum sensitivity, specificity and cut-off for the │ΔF_max_│ at each diagnostic threshold measured using histological analysis.

Criteria for microleakage severity	Cut-off value of │ΔF_max_│	Sensitivity	Specificity	AUC	95% CI
Score 0/1–3	10.10	0.82	0.91	0.91	0.87–0.96
Score 0–1/2–3	10.22	0.95	0.68	0.89	0.84–0.94
Score 0–2/3	15.21	0.82	0.73	0.83	0.76–0.89

AUC, area under the ROC curve; CI, confidence interval; score; dye penetration histological score.

## Discussion

The null hypothesis of this in vitro study was rejected because of the significant relationship between the fluorescence variable ΔF_max_ obtained using QLF technology and the actual depth of microleakage (µm) of the pit and fissure sealant. In this study, the QLF technology, based on the autofluorescence of teeth, was employed to objectively and quantitatively assess microleakage in pit and fissure sealants. The autofluorescence variable ΔF_max_ exhibited the highest level of accuracy for detecting initial microleakage, enabling the noninvasive identification of early-stage microleakage. Various fluorescence variables can be obtained during the evaluation of tooth autofluorescence using QLF technology. Among these, ΔF_max_, which reflects areas where tooth autofluorescence is most diminished and appears darkest, was selected for this study^50^. ΔF_max_ showed a significant increase in correlation with increasing histological scores at each stage, facilitating the differentiation of microleakage depth (Table 1, Fig. 1). Moreover, a strong significant correlation was observed between ΔF_max_ and histological scores (r = − 0.72, P = 0.001). According to previous studies, ΔF_max_ can be used to non-destructively evaluate early carious lesions progressing into the subsurface areas of pits and fissures on the occlusal surface of teeth^51,52^. Another study focusing on the detection of enamel cracks using QLF proposed ΔF_max_ as an indicator for lesion detection, as the fluorescence appears more scattered and darker in the deepest part of the lesion^53^. ΔF_max_, which represents the deepest part of the lesion, serves as a stable indicator for analyzing areas with tooth cracks, and it is less influenced by the selection of the area of interest (AOI)^53^. Based on these findings, ΔF_max_ is a reliable parameter for evaluating sealant microleakage, exhibiting low inter-examiner variability and minimal susceptibility to methodological influences.

Based on the evaluation of the diagnostic accuracy of QLF technology for detecting microleakage around pit and fissure sealants, the fluorescence variable obtained by using a QLF device was valid not only for determining the presence of microleakage but also for distinguishing its severity or progression level (AUC > 0.8). In particular, ΔF_max_ exhibited the highest level of diagnostic accuracy (AUC 0.91) when distinguishing between cases of no microleakage (score 0) and those of all microleakage levels (scores 1, 2, and 3), achieving both high sensitivity and specificity. Sealants typically lack fillers, allowing them to flow effectively into complex pits and fissure structures. Consequently, sealants exhibit relatively lower physical strength and are highly susceptible to microleakage. Therefore, the early detection of sealant microleakage is even more critical than that of other restorative materials. The effective ΔF_max_-based detection of sealant microleakage, ranging from initial enamel outer-half microleakage (score 1) to deeper levels (scores 2 and 3) described in this study, is of considerable clinical relevance.

When sealant microleakage extends beyond the inner half of the cross-sectional area (score 2 or 3), the risk of developing severe secondary caries increases. In this study, diagnosing microleakage beyond the inner half exhibited high diagnostic accuracy (AUC 0.91) and sensitivity (0.95) but moderate specificity (0.68). These results are consistent with those of previous systematic reviews that compared various methods for detecting secondary caries in amalgam and resin composite restorations^48^. In particular, when sealant microleakage extends beyond the inner half, this suggests that bacteria have already penetrated the sealant base, increasing the risk of partial sealant loss or the onset of secondary caries. In such clinical scenarios where sealant reapplication can be performed without additional tooth removal, diagnostic methods such as QLF, which offer higher sensitivity than specificity, are deemed to have notable clinical utility from a preventive standpoint.

The resin components comprising the sealant typically contain radiopaque fillers to facilitate their identification on dental radiographs. The types and quantities of these fillers may vary depending on the manufacturer. In this study, Clinpro sealant was used. Upon application and curing, the sealant turned an opaque off-white color when visually observed and appeared darker under fluorescent observation compared to the natural tooth enamel (Fig. 1). In a previous study using QLF to evaluate various types of resin composites, significant differences were observed in their autofluorescence patterns. Most resin composites exhibit brighter fluorescence than that of natural teeth. However, some products displayed darker fluorescence than tooth autofluorescence. This difference in fluorescence is believed to originate from variations in the fluorescent components of the products^54^. The dark fluorescence of the sealant material used in the current study may have interfered with the detection of fluorescence loss due to microleakage, potentially affecting the interpretation of the results in some cases. However, as reported in a previous study, using a sealant that exhibits brighter fluorescence than natural tooth enamel may enable a more sensitive evaluation of fluorescence loss associated with microleakage^54^.

This study demonstrated the effectiveness of using QLF to detect microleakage resulting from improper sealant application and quantify its depth. However, a limitation of this study is that we did not monitor whether sealant repair was necessary in teeth with microleakage. QLF technology allows for simultaneous assessment of changes in both tooth autofluorescence and red fluorescence produced by bacterial metabolites. However, because this study used extracted teeth, it was challenging to evaluate the impact of red fluorescence originating from actual oral biofilm. In an oral environment, red fluorescence from bacterial biofilms infiltrating the areas of sealant or restoration microleakage can influence fluorescence changes. Therefore, future research should consider evaluating not only the fluorescence changes caused by actual microleakage in the oral environment but also the fluorescence changes associated with bacteria-related microleakage. In future clinical studies, it will be necessary to observe and track the occurrence of microleakage using QLF to evaluate subtle changes over time.

## Conclusions

Utilizing QLF technology, our study has demonstrated a significant relationship between the QLF variable ΔF_max_ and the depth of microleakage in pit and fissure sealants. Notably, this technology exhibits a very high diagnostic capability in detecting leakage within the outer half of the sealant. This confirms the utility of QLF in noninvasively detecting and evaluating the severity of sealant microleakage, offering a valuable tool for enhancing sealant retention and preventive dental care. The application of QLF technology into routine dental practices may facilitate the effective management and monitoring of sealants, thereby offering a viable strategy for the effective prevention of secondary caries.

## Methods

### Study design

**Figure 2:**
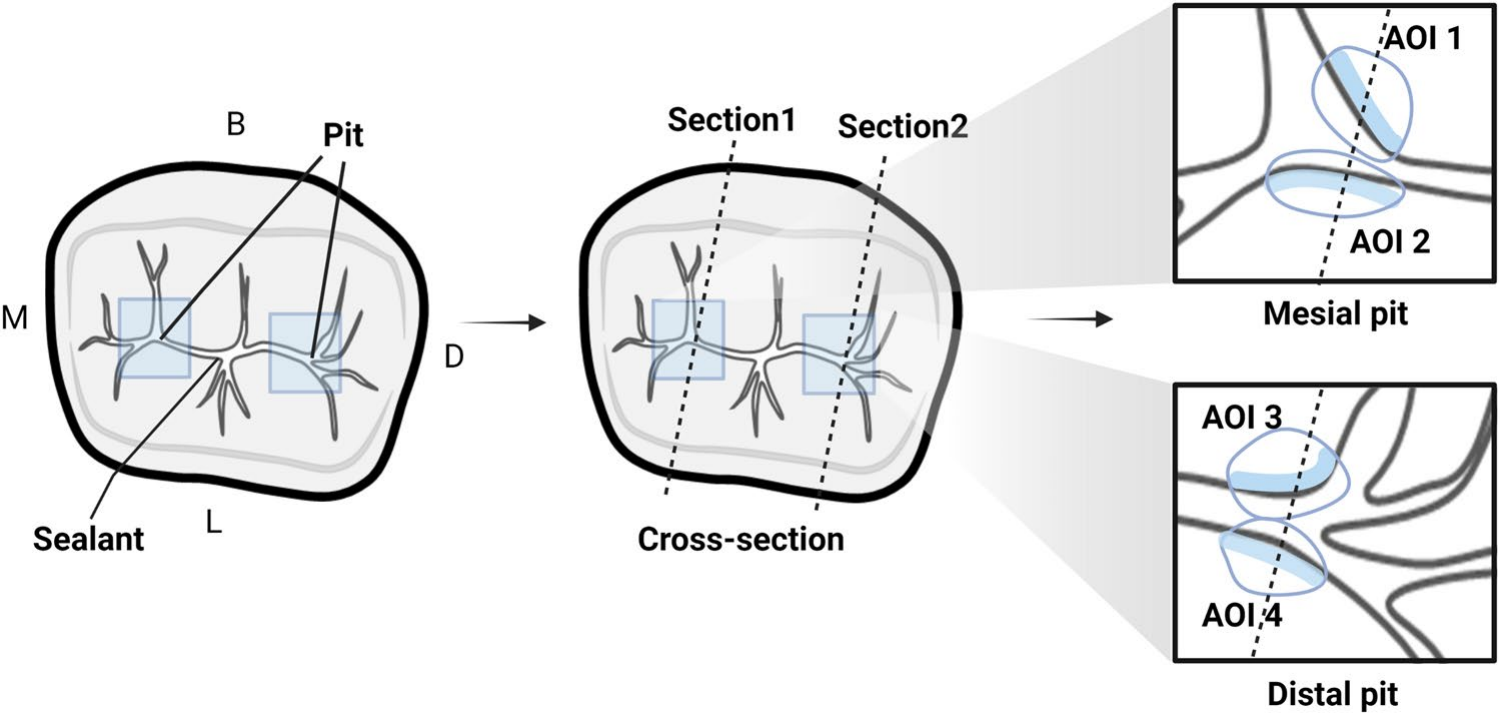
Occlusal surface areas of interest (AOIs) of the sealant. The total number of AOIs is four. The selection of AOIs on the occlusal surface was based on the central groove branching buccally or lingually from the mesial and distal pits. B: buccal surface; L: lingual surface; M: mesial surface; D: distal surface.

This study followed previously described methods for artificially inducing microleakage in a laboratory setting^55^. To create microleakage of sound sealants at various depths, two application conditions were used: the method recommended by the manufacturer and the saliva contamination method. Prior to sealant application on all teeth, a standardized pretreatment procedure was carried out, followed by drying. Sealant application according to the manufacturer’s instructions (number of teeth = 18) or saliva contamination (number of teeth = 22) was implemented. Subsequently, artificial hydration was performed to replicate the conditions within the oral cavity after pit and fissure sealant application in all 40 samples. Following the hydration process, four evaluation areas (number of surfaces = 160) on each tooth were selected as AOIs, denoted as AOI 1–4 (Fig. 2). The selection of AOIs was limited to the area of supplemental grooves on the occlusal surface. The AOIs were located along the central groove, branching in the buccal or lingual direction from both the mesial and distal pits for evaluation. Each tooth was captured using QLF technology from the occlusal direction, followed by dye penetration. Cross-sectioning was performed immediately thereafter for histological analysis.

### Preparation of the specimens

The use of extracted teeth and human saliva in this study was approved by the Institutional Review Board of Yonsei University Dental Hospital (IRB number: 2–2017–0044, date: Aug 31, 2017). We adhered to the STROBE guidelines for reporting the results. All procedures involving extracted teeth and saliva were performed in accordance with the 1964 Declaration of Helsinki.

Participants were informed of the study, and informed consent was obtained. The extracted sound teeth were frozen at − 20 °C. A total of 44 sound permanent molars without dental caries or discoloration were used after the removal of dental plaque. The remaining deposits attached to the tooth surface were removed using a manual scaler (Anterior Sickle Scaler; Hu-Friedy, Chicago, IL, USA), and a bristle brush (ICB brush; Micron, Korea) was mounted on a low-speed handpiece (Handpiece; Lasungmedice, Incheon, Korea) to remove the plaque on the tooth with fluoride-free pumice. The roots were then cut using cutting equipment (Diamond discs; NTI-Kahla, Kahla, Germany) and molded using resin on an acrylic block (15 mm × 12 mm × 6 mm; Ortho-Jet; Lang Dental, Wheeling, IL, USA).

According to the manufacturer’s guidelines, the standardized method was employed to apply sealant to 18 teeth as follows: The pits and fissures of the teeth were etched for 15 s using a 35% phosphoric acid etchant (Scotchbond etchant; 3M-ESPE, St Paul, MN, USA), washed with water for 10 s, and dried. Clinpro (Clinpro sealant; 3M-ESPE, St Paul, MN, USA) was used as the pit and fissure sealant material, and an LED curing unit (Dr’s Light; Good Doctors, Incheon, Korea) was used for light curing. The principal ingredients of Clinpro sealant are triethylene glycol dimethacrylate, bisphenol A-glycidyl methacrylate, tetrabutylammonium tetrafluoroborate, dichloride methylsilane, silica, and dye. The wavelength range of the light source was 440–490 nm. In accordance with a previous study, the teeth were light-cured for 40 s^56^. The tip of the light was held as close as possible to the sealant, according to the manufacturer’s instructions.

For the remaining 22 teeth, the method from a previous study was utilized to induce microleakage around the pit and fissure sealant^56^. Although the etching process and rinsing of the etchant material were conducted according to the manufacturer’s guidelines, saliva was applied immediately before applying the sealant to induce incomplete penetration of the sealant and promote microleakage formation. The saliva contamination method involved applying human saliva (0.1 ml) to the occlusal surface, followed by the absorption of excessive saliva using a science wiper (Kimtech Science wiper; Yuhan-Kimberly, Seoul, Korea). After the application of the sealant, the instruments and application methods used were implemented following the same procedures as those in the manufacturer’s instructions group. All the specimens processed with either of the two different methods of sealant application (manufacturer’s instructions or saliva contamination) underwent an artificial hydration process for 48 h by immersion in distilled water (JW Pharmaceutical, Seoul, Korea). This process aimed to create an environment resembling the oral cavity while accelerating microleakage conditions.

### Acquisition of fluorescence images

In this study, the microleakage of sealants was assessed non-destructively by capturing fluorescent images from the occlusal surface of each tooth. Fluorescence imaging was performed at a distance of 10 cm from the occlusal surface using a quantitative light-induced fluorescence-digital system (QLF-Biluminator; Inspektor Research Systems, Amsterdam, The Netherlands) in a dark room environment. The QLF-D is a single-lens reflex camera with two light-emitting diode lamps mounted in front of a 60-mm macro lens. A total of 12 blue lights (wavelength 405 nm) and four white lights were attached, and each light source was irradiated. QLF-D continuously captured white-light and blue-light images under the same conditions. The QLF-D photographic conditions were as follows: shutter speed of 1/13, aperture value of 14, ISO 250, and fluorescence image: shutter speed of 1/50, aperture value of 10, and ISO 1600.

### Quantitative evaluation of autofluorescence

Fluorescence analysis was conducted using fluorescent images captured from the occlusal direction prior to histological evaluation. All images were independently analyzed by two trained examiners (S.M. and K.M.). The two examiners standardized the analysis area before the evaluations and then quantified the autofluorescence of the microleakages detected in the pit and fissure sealant margins using a specific program (QA2 v1.26; Inspektor Research Systems, Amsterdam, The Netherlands). In cases of disagreement, the two examiners reached an agreement through discussion. When the sound part was selected as a patch in the pit margins of the sealant, the microleakage site showed a loss of autofluorescence. The maximum fluorescence loss, which represented the largest amount of fluorescence loss, was calculated as ΔF_max_ [%] (Fig. 1). The delta F_max_, which indicates the maximum fluorescence loss, refers to the darkest area where fluorescence is reduced and is reported to reflect the deepest point within that area histologically^53^. The two examiners (S.M. and K.M.) conducted fluorescence analysis prior to the histological assessment of microleakage severity, performing the analysis in a blinded manner without prior information about the extent of microleakage.

### Histological analysis

The teeth were immersed in a 1% methylene blue solution (vista-blue methylene blue dye; Vista Apex, WI, USA) for 24 h to facilitate penetration of the solution between the sealant and occlusal surface. Nail varnish was applied to the occlusal surface, except for 1 mm on the margin of the sealant, and was dried to prevent unnecessary methylene blue solution penetration from the surface. The cross-section of one pit was marked with a water-erasable pen on an acrylic block molded with a crown to analyze the mesial and distal pits and cut at the same site for histological evaluation. After each specimen was washed and dried under water, the pits were sectioned longitudinally in the buccolingual direction using a cutter (TechCut 4; Allied High Tech Products, CA, USA). Four cut sections were obtained per tooth for each pit (Fig. 2). The mesial and distal pits of each tooth were marked by selecting a higher score for solution penetration between the same two tooth surfaces when viewed from the buccolingual direction, and four histological scores per tooth were obtained (Fig. 2). The microleakage of the exposed cut surface and the degree of penetration of the dye were observed using an optical microscope (Axio Imager A1; Carl Zeiss, Oberkochen, Germany) at a magnification of 50×. The selection of AOIs was limited to the area of supplemental grooves on the occlusal surface, and a total of four AOIs were designated. These AOIs were located along the central groove, branching out in the buccal or lingual direction from both the mesial and distal pits for evaluation.

**Figure 3:**
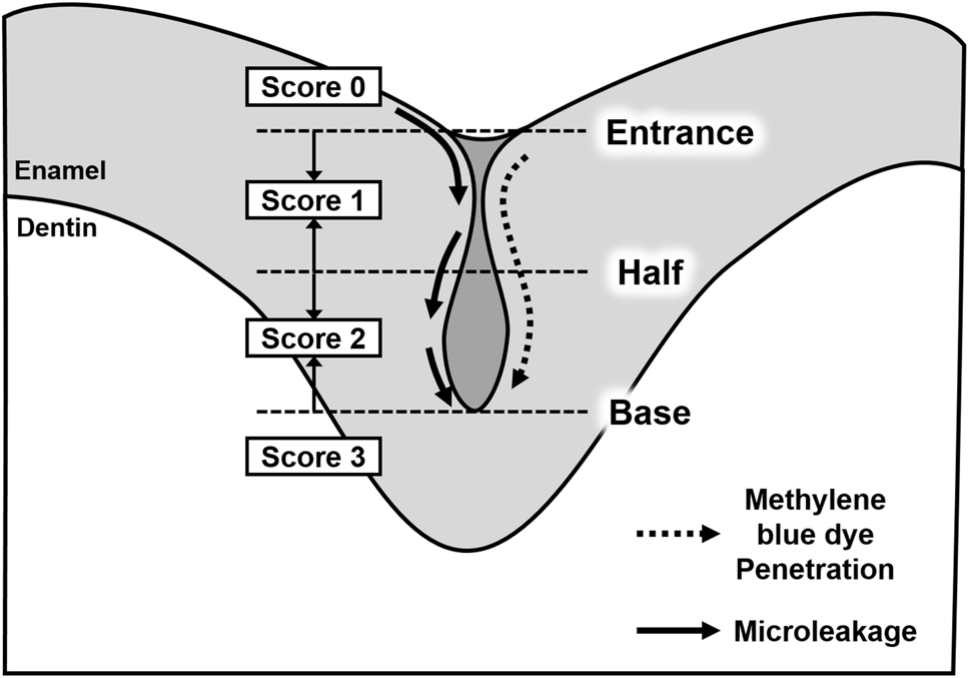
Schematic diagram of a cross-sectioned specimen used for determining the histological score of dye penetration into microleakage.

The dye penetration score for microleakages in the pit and fissure sealants was evaluated according to the classification system used in a previous study (Fig. 3)^57^. Score 0: no dye penetration; score 1: dye penetration restricted to the outer half of the pit and fissure sealant; score 2: dye penetration to the inner half of the pit and fissure sealant; and score 3: dye penetration into the underlying pit and fissure. The severity of the microleakage was scored based on the depth of dye penetration in the cross-sectioned samples, and these scores were compared with the maximum fluorescence loss.

### Statistical analysis

The correlation between histological results and fluorescence parameter values was analyzed using Spearman’s rank correlation. The sensitivity, specificity, and area under the receiver operating characteristic curve of the fluorescence variable were analyzed to evaluate the validity of the fluorescence variable ΔF_max_ to detect the histological classification score. A significance level of 5% was adopted for all analyses, and the data were analyzed using R, version 4.0.2 (R; R Core Team, Vienna, Austria).

## Data Availability

The datasets used and/or analyzed during the current study are available from the corresponding author upon reasonable request.
